# Supplementary material for: High-resolution cryo-EM structure of the Pseudomonas bacteriophage E217
Source: Nat Commun. 2023 Jul 8;14:4052. doi: 10.1038/s41467-023-39756-z (PMC10329688; doi:10.1038/s41467-023-39756-z)
Supplement: Supplementary file 1 — Supplementary Information [file 41467_2023_39756_MOESM1_ESM.pdf]

High-resolution cryo-EM structure of the *Pseudomonas* bacteriophage E217

Fenglin Li [1¥], Chun-Feng David Hou [1¥], Ravi K Lokareddy [1], Ruoyu Yang [1], Francesca Forti [2], Federica Briani [2 †] and Gino Cingolani [1 †]

[1] Department of Biochemistry and Molecular Biology, Thomas Jefferson University, 1020 Locust Street, Philadelphia, PA 19107, USA.

[2] Dipartimento di Bioscienze, Università degli Studi di Milano, Milan, Italy

[¥] these authors contributed equally

[†] Corresponding authors: E-mail: federica.briani@unimi.it; gino.cingolani@jefferson.edu

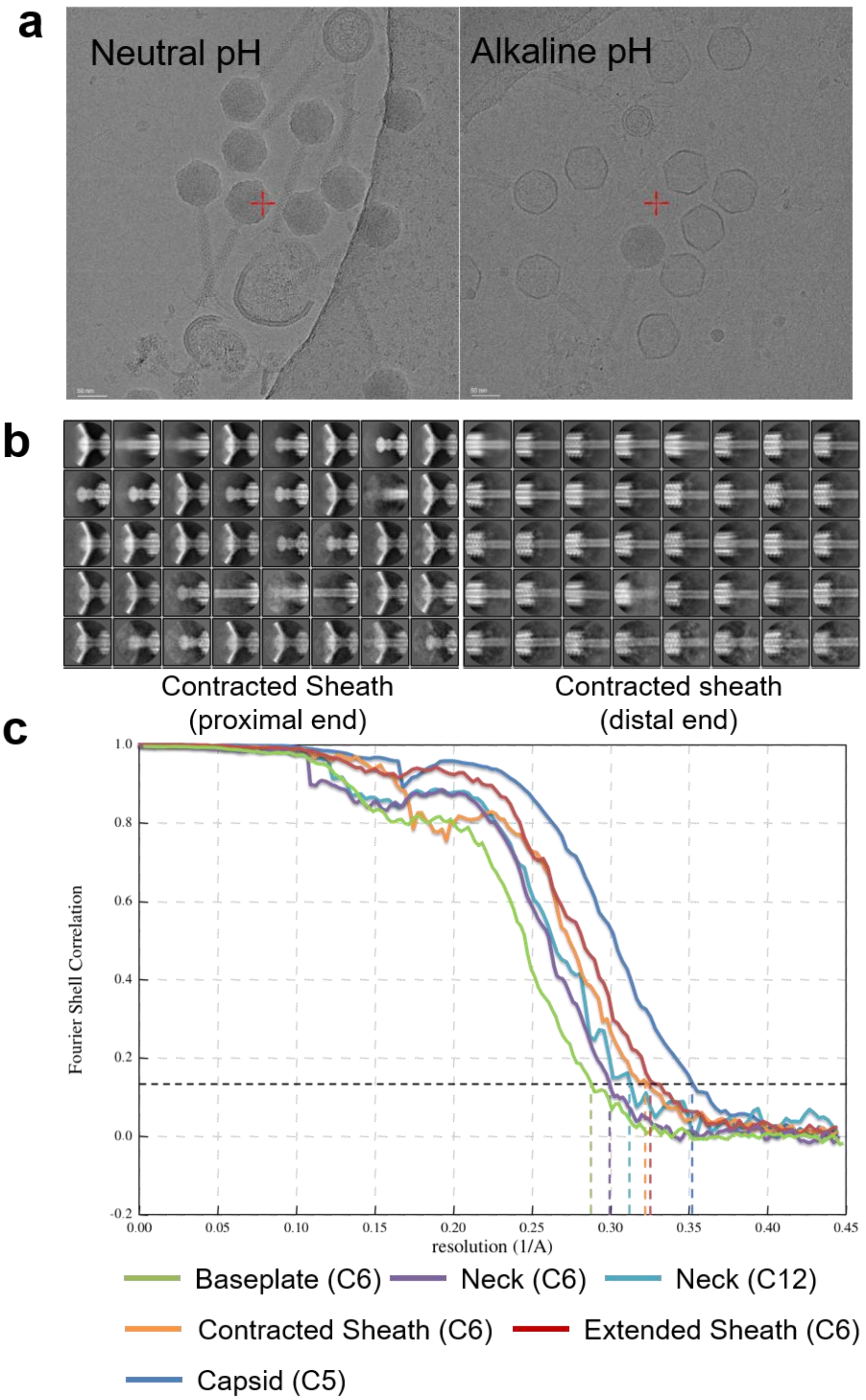

Supplementary Figure 1. Cryo-EM methods. (**a**) Representative cryo-micrographs of E217 at neutral and alkaline pH selected from 22,015 and 22,714 micrographs, respectively. (**b**) 2D classes of neck and sheath regions in the post-ejection conformation. (**c**) FSC resolution curves for all reconstructions presented in this paper. The cut-off is 0.143.

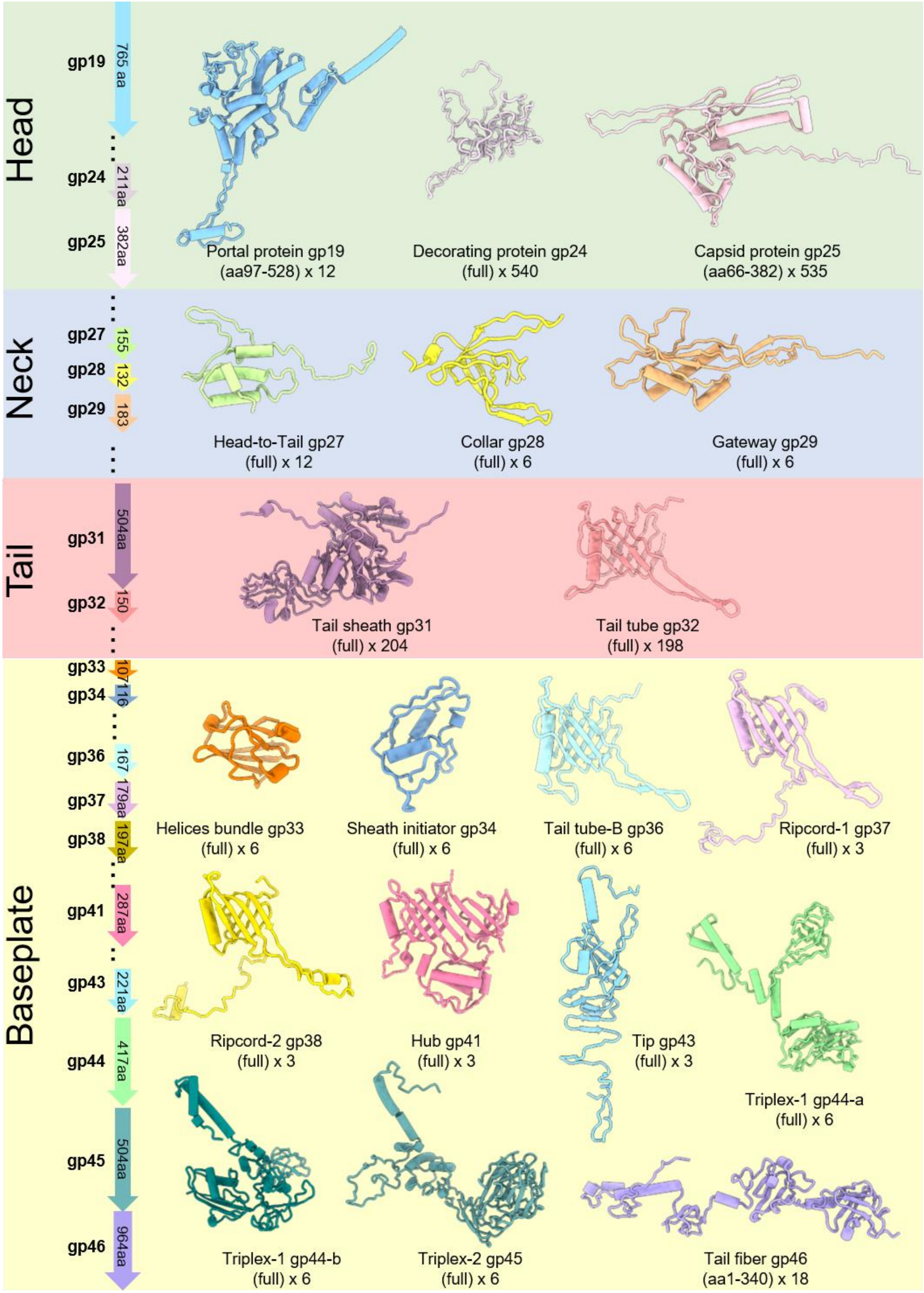

Supplementary Figure 2. Atlas of all protein structures built in this paper.

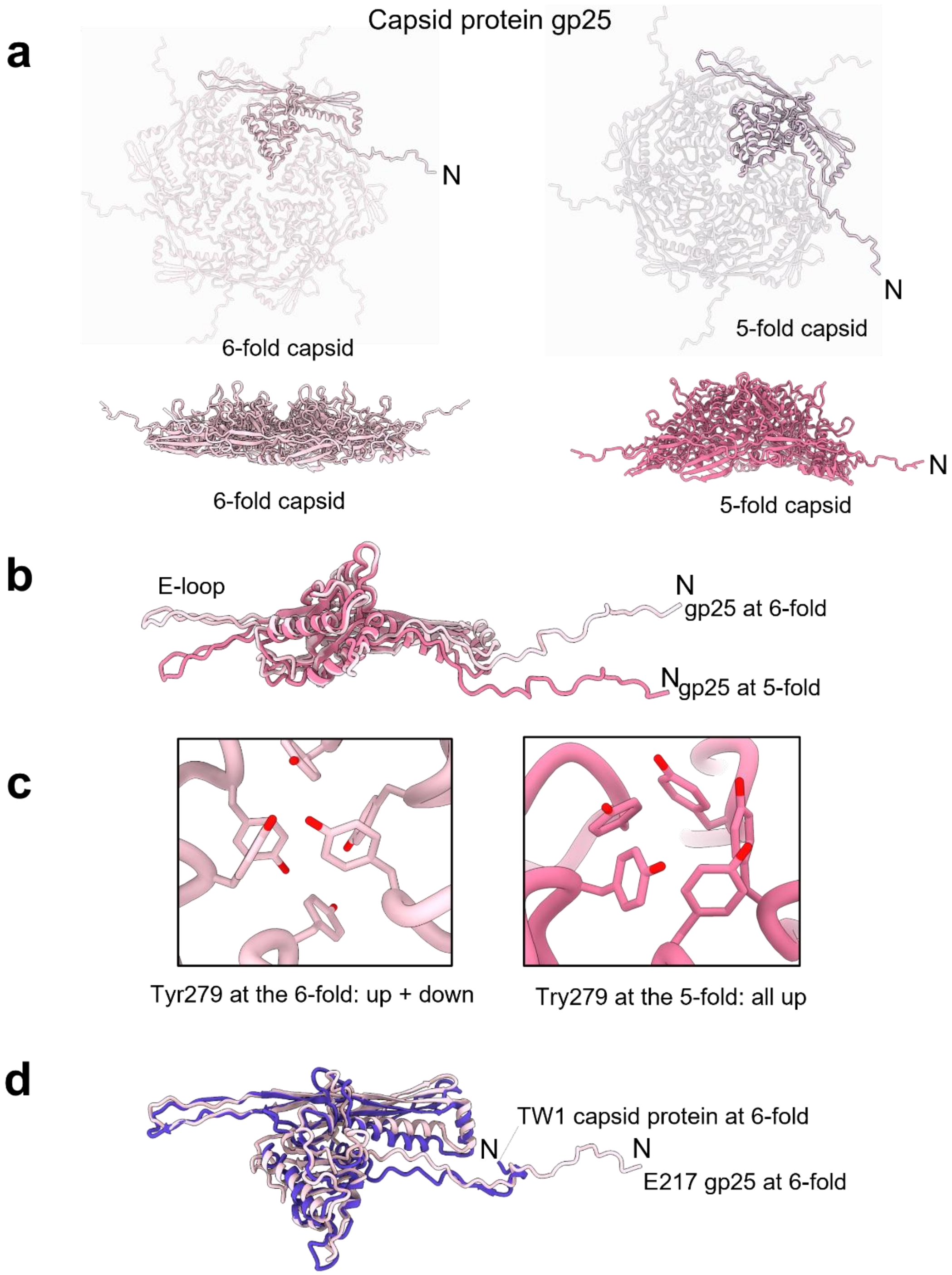

Supplementary Figure 3. Structure and plasticity of the E217 capsid protein gp25. Assembly of gp25 in (**a**) hexamers (6-fold) and pentamers (5-fold). (**b**) The RMSD between gp25 *quasi*-equivalent subunits at 5- and 6-fold is 5.7 Å for all residues and 2.3 Å for the gp25 core (e.g., deleting the E-loop and N-terminus). (**c**) Tyr279 at the 6-fold and 5-fold vertices adopt different side chain orientations. (**d**) Superimposition of E217 gp25 and TW1 capsid protein found at the 6-fold vertex. The RMSD is 8.8 Å for 274 aligned residues out of TW1 317 total residues.

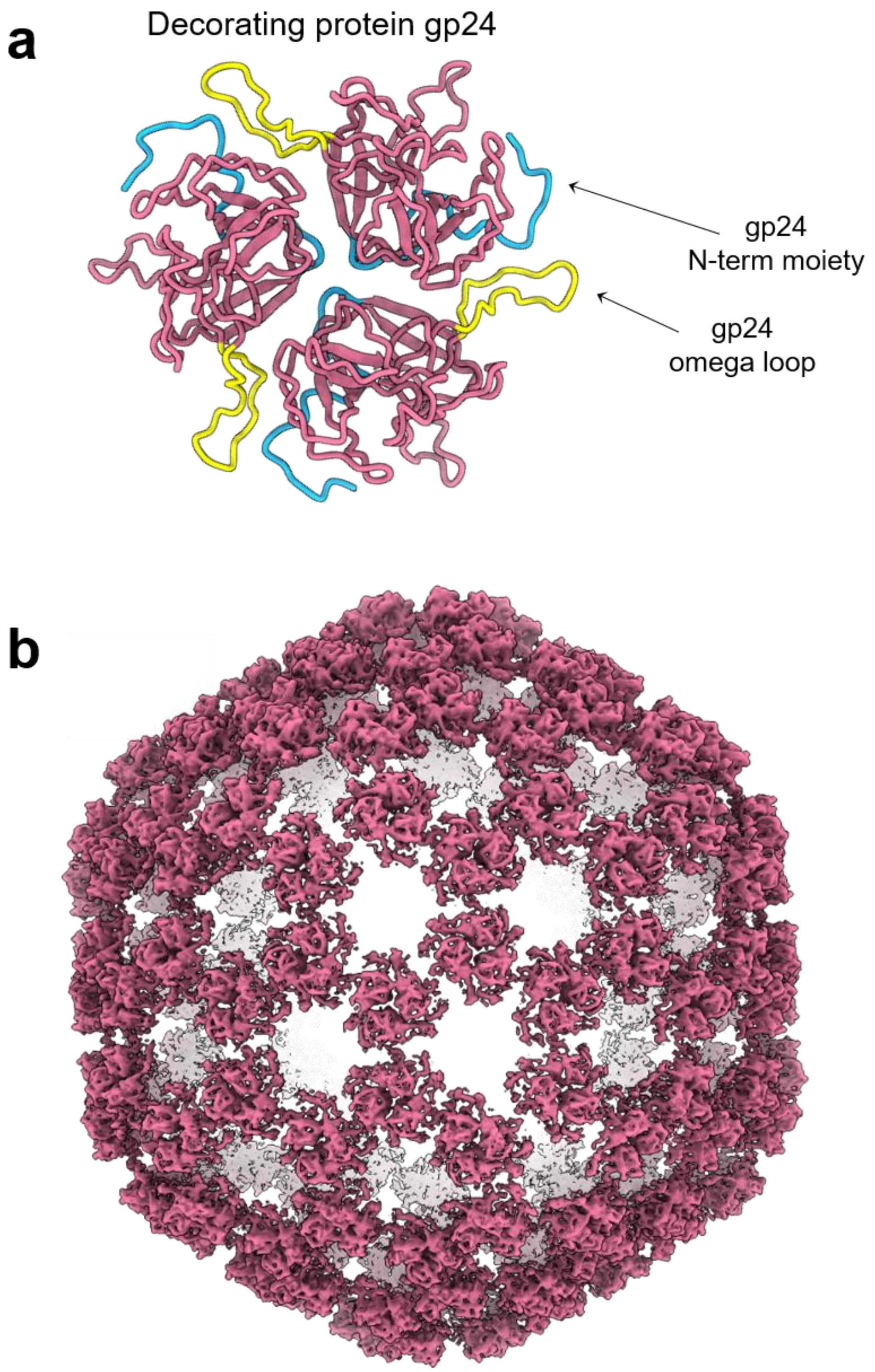

Supplementary Figure 4. Organization of the decorating protein gp24.(**a**) Ribbon diagram of the gp24 trimer with the N-terminal moiety and omega loop colored blue and yellow, respectively. (**b**) Cage formed by all gp24 trimers decorating the E217 mature head.

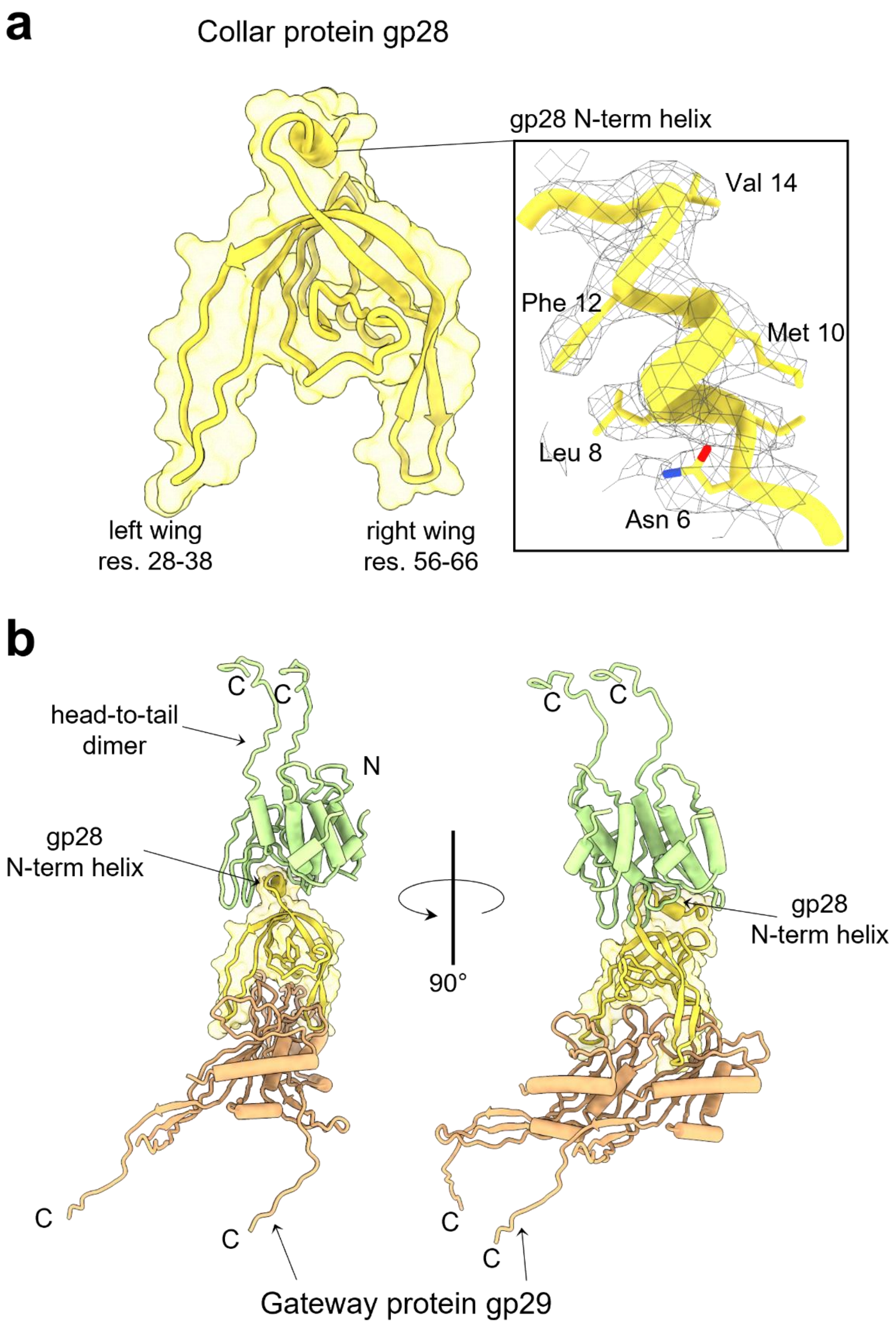

Supplementary Figure 5. Neck proteins gp28 and gp29. (**a**) The tertiary structure of the collar protein gp28 is shown as a ribbon overlaid to a semitransparent yellow solvent surface. Representative C12 density map contoured at 3.8 σ and overlaid to the refined gp28 model (residues 6-14). (**b**) Ribbon diagram of how gp28 bridges a dimer of head-to-tail subunits (green) to the gateway protein (orange).

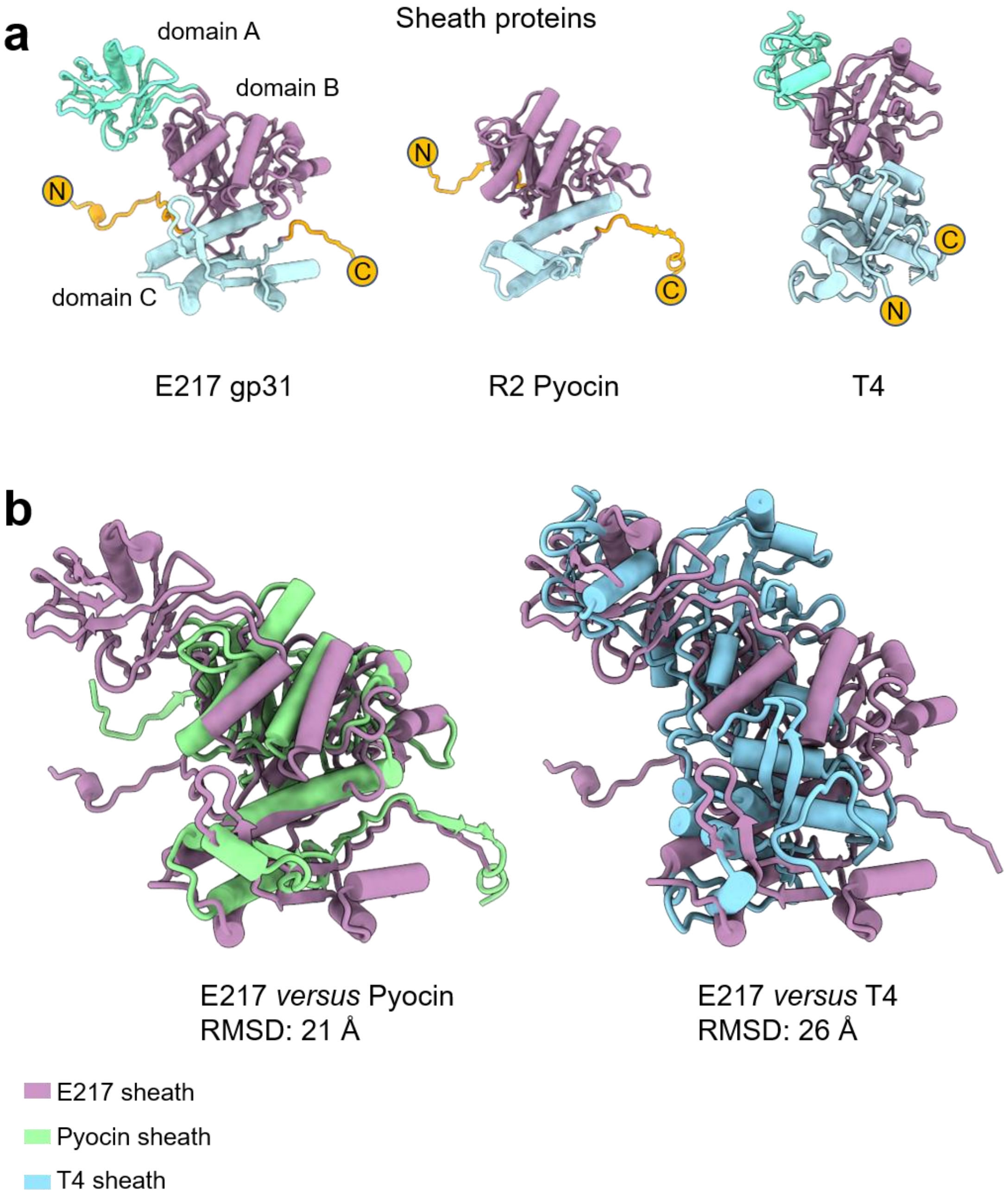

Supplementary Figure 6. Structure and conservation of the sheath protein gp31.

(**a**) Three-dimensional structure of the sheath proteins from phage E217, R2 pyocin, and phage T4 color-coded by domains. (**b**) Structural superimposition of the E217 sheath protein (purple) with the counterparts from the R2 pyocin (green) and phage T4 (cyan). The RMSD between E217 and pyocin sheath proteins is 21 Å for 39 aligned Cα atoms out of 504 and 385 Cαs, respectively. Instead, the RMSD between E217 and T4 sheath proteins is 26 Å with only 5 aligned Cα atoms out of 504 and 479 Cαs, respectively. After removing domain A from the E217 sheath protein, the RMSD between E217 and pyocin sheaths drops to 16 Å with 295 aligned Cα atoms but is still 26 Å for E217 and T4 sheaths (for 274 aligned Cα atoms).

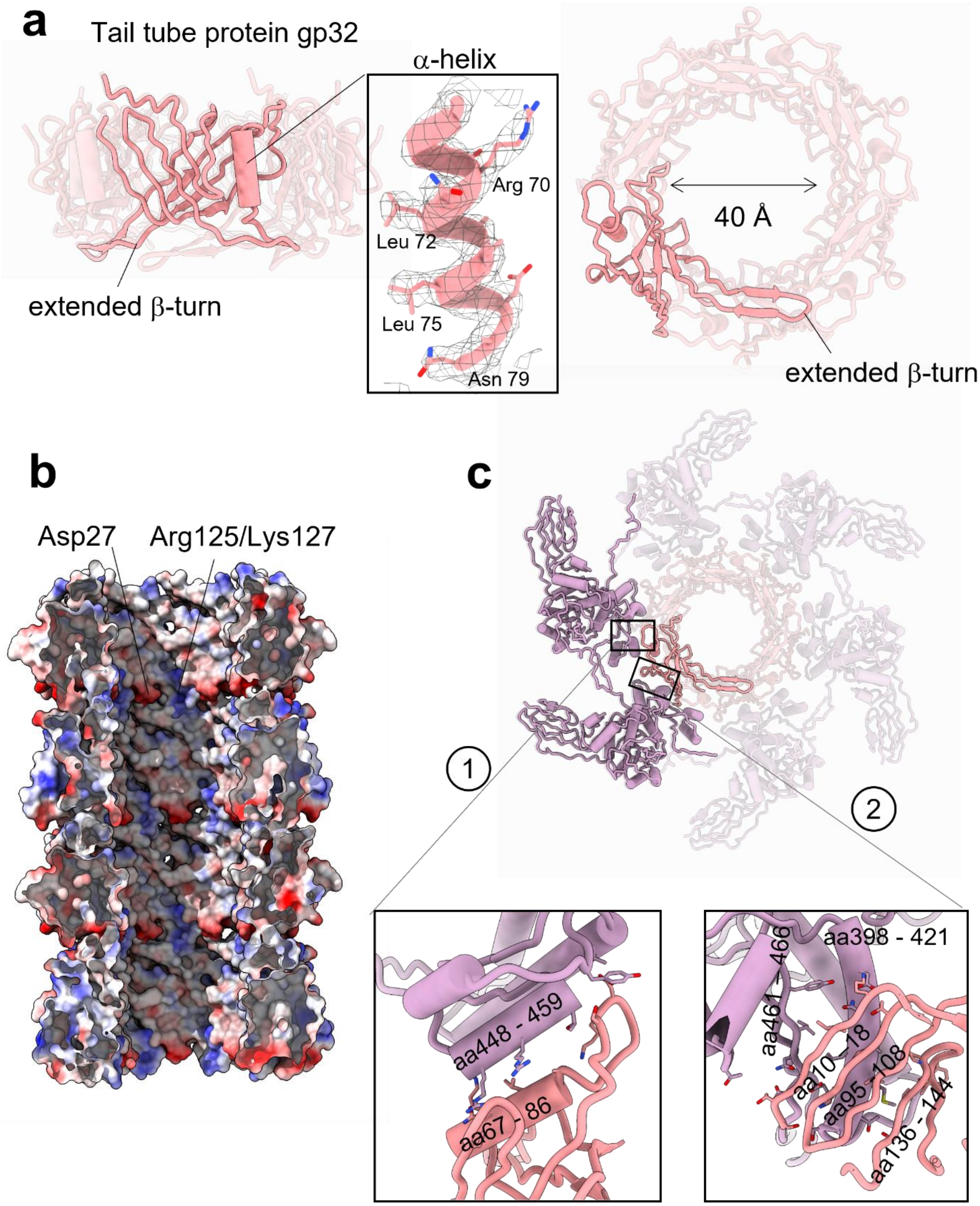

Supplementary Figure 7. Structure and assembly of the E217 tail tube gp32. (**a**) Ribbon diagram of the tail tube hexamer with only one gp32 subunit colored in pi nk. Representative C6 cryo-EM electron density map contoured at 4.0 σ and overlaid to the refined gp32 model (residues 68-79) (**b**) The electrostatic potential surface inside the tail tube channel. (**c**) Contacts between tail tube hexamers and sheath proteins in the extended conformation.

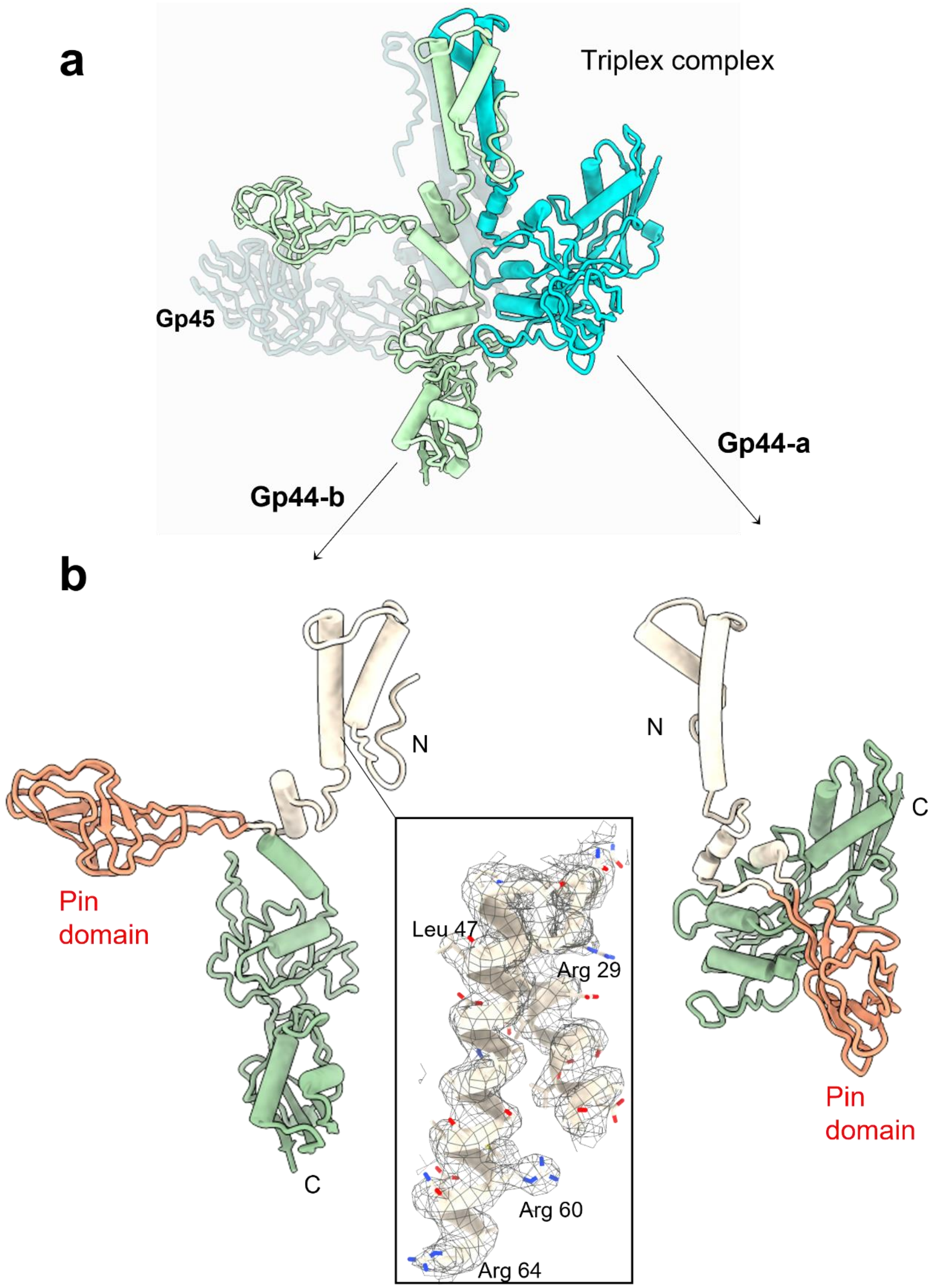

Supplementary Figure 8. The architecture of the E217 triplex complex. (**a**) The triplex complex with gp44-a and gp44-b is colored in cyan and light green, respectively. (**b**) Individual structures of gp44-a and gp44-b are colored by domains. Representative C3 density map contoured at 3.3 σ and overlaid to the refined gp44-a model (residues 16-64).

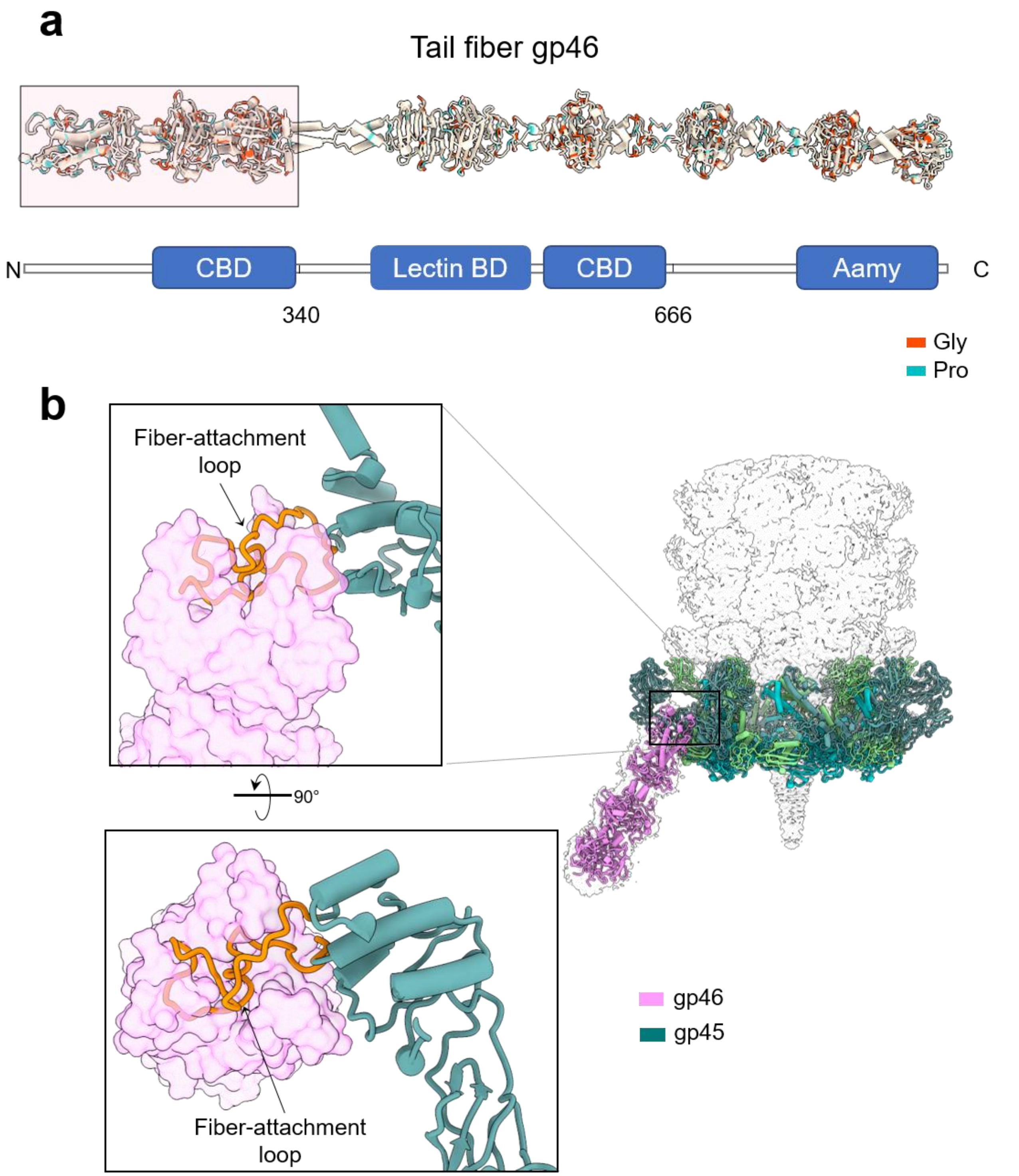

Supplementary Figure 9. Tail fiber gp46 structure and attachment to the baseplate
(**a**) An AlphaFold2 model of the full-length tail fiber gp46. Only the N-terminal 340 residues were modeled in the density. Predicted functional domains are shown as blue boxes (CBD = Carbohydrate Binding Domain, Aamy = Alpha-amylase domain). (**b**) Association of gp46 N-termini with the triplex complex subunit gp45.

Supplementary Table 1. Summary of all binding interfaces, bonding, and energetics in E217 extended tail determined using PISA.

SYMMETRY MISMATCHED INTERFACE (COPY NUMBER RATIO)	HEAD-TO-TAIL: COLLAR (2 : 1)	TAIL TUBE-B : RIPCORD-1 (2 : 1)	TAIL TUBE-B : RIPCORD-2 (2 : 1)	TRIPLEX: TAIL-FIBER (1 : 3)
No. of interface residues	2:4	8:12	8:13	39:37
Interface areas (Å^2)	303:275	555:523	623:588	1833:1808
No. of salt bridges	-	-	-	-
No. of hydrogen Bonds	-	3	3	9
No. of non-bonded Contacts	18	98	78	1091
