# Supplementary material for: High-resolution cryo-EM structure of the Pseudomonas bacteriophage E217
Source: Nat Commun. 2023 Jul 8;14:4052. doi: 10.1038/s41467-023-39756-z (PMC10329688; doi:10.1038/s41467-023-39756-z)
Supplement: Supplementary file 3 — Reporting Summary [file 41467_2023_39756_MOESM3_ESM.pdf]

nature portfolio

Corresponding author(s): Gino Cingolani

Last updated by author(s): Jun 7, 2023

Reporting Summary

Nature Portfolio wishes to improve the reproducibility of the work that we publish. This form provides structure for consistency and transparency in reporting. For further information on Nature Portfolio policies, see our Editorial Policies and the Editorial Policy Checklist.

Statistics

For all statistical analyses, confirm that the following items are present in the figure legend, table legend, main text, or Methods section.

n/a	Confirmed	
☐	☒	The exact sample size (*n*) for each experimental group/condition, given as a discrete number and unit of measurement
☐	☒	A statement on whether measurements were taken from distinct samples or whether the same sample was measured repeatedly
☐	☒	The statistical test(s) used AND whether they are one- or two-sided<br>*Only common tests should be described solely by name; describe more complex techniques in the Methods section.*
☒	☐	A description of all covariates tested
☒	☐	A description of any assumptions or corrections, such as tests of normality and adjustment for multiple comparisons
☐	☒	A full description of the statistical parameters including central tendency (e.g. means) or other basic estimates (e.g. regression coefficient) AND variation (e.g. standard deviation) or associated estimates of uncertainty (e.g. confidence intervals)
☐	☒	For null hypothesis testing, the test statistic (e.g. *F*, *t*, *r*) with confidence intervals, effect sizes, degrees of freedom and *P* value noted<br>*Give P values as exact values whenever suitable.*
☒	☐	For Bayesian analysis, information on the choice of priors and Markov chain Monte Carlo settings
☒	☐	For hierarchical and complex designs, identification of the appropriate level for tests and full reporting of outcomes
☒	☐	Estimates of effect sizes (e.g. Cohen's *d*, Pearson's *r*), indicating how they were calculated

Our web collection on statistics for biologists contains articles on many of the points above.

Software and code

Policy information about availability of computer code

Data collection	Cryo-EM: TEM interface (Talos v7.x, TEM v7.X, TIA v5.X, FluCam Viewer v7.X) and EPU (v2.X).
Data analysis	Cryo-EM Image Analysis Software: MontionCor2(v1.X), CTFFIND (v4.X), RELION (v3.X), Scipion (v3.X); Atomic modeling and visualization: Coot (v0.8.9), Chimera (v1.X), ChimeraX (v1.X), Phenix (v1.X), PyMOL (v2.X); Atomic model analysis web services: DALI server (http://ekhidna2.biocenter.helsinki.fi/dali/), PDBePISA (https://www.ebi.ac.uk/pdbe/pisa/), PDBsum (http://www.ebi.ac.uk/thornton-srv/databases/pdbsum/), SMART (http://smart.embl-heidelberg.de/), SuperPose 1.0 (http://superpose.wishartlab.com/).

For manuscripts utilizing custom algorithms or software that are central to the research but not yet described in published literature, software must be made available to editors and reviewers. We strongly encourage code deposition in a community repository (e.g. GitHub). See the Nature Portfolio guidelines for submitting code & software for further information.

Data

Policy information about availability of data

All manuscripts must include a data availability statement. This statement should provide the following information, where applicable:

- Accession codes, unique identifiers, or web links for publicly available datasets
- A description of any restrictions on data availability
- For clinical datasets or third party data, please ensure that the statement adheres to our policy

Entry codes for Protein Data Bank (https://www.rcsb.org/) are 8FRS [http://doi.org/10.2210/pdb8frs/pdb], 8FVH [http://doi.org/10.2210/pdb8fvh/pdb], 8FUV

[http://doi.org/10.2210/pdb8fuv/pdb], 8FVG [http://doi.org/10.2210/pdb8fvg/pdb], and 8EON [http://doi.org/10.2210/pdb8eon/pdb],
For Electron Microscopy Data Bank (https://www.ebi.ac.uk/emdb/) are EMD-29406 [http://www.ebi.ac.uk/emdb/EMD-29406], EMD-29487 [http://www.ebi.ac.uk/emdb/EMD-29487], EMD-29481 [http://www.ebi.ac.uk/emdb/EMD-29481], EMD-29486 [http://www.ebi.ac.uk/emdb/EMD-29486], and EMD-28405 [http://www.ebi.ac.uk/emdb/EMD-28405].

Research involving human participants, their data, or biological material

Policy information about studies with human participants or human data. See also policy information about sex, gender (identity/presentation), and sexual orientation and race, ethnicity and racism.

Reporting on sex and gender	Not applicable.
Reporting on race, ethnicity, or other socially relevant groupings	Not applicable.
Population characteristics	Not applicable.
Recruitment	Not applicable.
Ethics oversight	Not applicable.

Note that full information on the approval of the study protocol must also be provided in the manuscript.

Field-specific reporting

Please select the one below that is the best fit for your research. If you are not sure, read the appropriate sections before making your selection.

☒ Life sciences ☐ Behavioural & social sciences ☐ Ecological, evolutionary & environmental sciences

For a reference copy of the document with all sections, see nature.com/documents/nr-reporting-summary-flat.pdf

Life sciences study design

All studies must disclose on these points even when the disclosure is negative.

Sample size	The sample size for the phage studied in this paper was calculated using a plaque-forming unit (PFU) assay.<br>For cryo-electron microscopy (cryo-EM) studies, the sample size was determined by counting single particles in micrographs with an automated particle counter in RELION. We collected 22,015 and 22,714 micrographs for the extended and contracted E217, respectively.<br>A total of 13,302, 15,505, 13,257, 10,8269 and 10,126 particles were used for the 5 reconstructions reported in this paper, corresponding to EMD-29406, EMD-29487, EMD-29481, EMD-29486, and EMD-28405, respectively. For all five density maps, the sample size was deemed sufficient based on the high quality of the final map and the shape of the relative FSC curves.
Data exclusions	No data were excluded for LPS binding assay.<br>Cryo-EM: Single particle image data were excluded based on quality (CTF estimation) to eliminate lower resolution micrographs, and 2D/3D classification to eliminate smeared, broken, or heterogeneous particles.
Replication	For LPS binding assay, each group was repeated four times. Each repeat was successful.<br>For single particle analysis, each reconstruction was repeated at least three times. Each reconstruction attempt yielded the same final density map.
Randomization	For LPS binding assay, cells were randomly allocated into different experimental groups.<br>Cryo-EM: to compute Fourier-Shell Correlation (FSC) resolution, 2D data are randomly split into non-overlapping half-sets and processed identically. The FSC resolution was estimated at CC=0.143 (Gold Standard).
Blinding	Blinding was not relevant to the study, since no animals nor patients were involved.

Reporting for specific materials, systems and methods

We require information from authors about some types of materials, experimental systems and methods used in many studies. Here, indicate whether each material, system or method listed is relevant to your study. If you are not sure if a list item applies to your research, read the appropriate section before selecting a response.

Materials & experimental systems

n/a	Involved in the study
☒	☐ Antibodies
☒	☐ Eukaryotic cell lines
☒	☐ Palaeontology and archaeology
☒	☐ Animals and other organisms
☒	☐ Clinical data
☒	☐ Dual use research of concern
☒	☐ Plants

Methods

n/a	Involved in the study
☒	☐ ChIP-seq
☒	☐ Flow cytometry
☒	☐ MRI-based neuroimaging
